# A primer on sleep neuroscience for psychiatry

**DOI:** 10.1038/s44277-026-00055-2

**Published:** 2026-02-17

**Authors:** Jared M. Saletin, Giulia R. Righi

**Affiliations:** 1 Department of Psychiatry and Human Behavior, Alpert Medical School of Brown University, Providence, RI USA; 2 COBRE Center for Sleep and Circadian Rhythms in Child and Adolescent Mental Health, Emma Pendleton Bradley Hospital, East Providence, RI USA

**Keywords:** Human behaviour, Translational research

Sleep problems are common in psychiatric and neurodevelopmental disorders, potentially emerging before other symptoms. The neuroscience of sleep offers a rich lens through which novel biomarkers and innovative treatments can be found. Researchers and clinicians alike will benefit from a deeper understanding of the role of sleep in psychiatric disorders.

Sleep neurophysiology is typically studied with at-home or laboratory polysomnography: electroencephalography (EEG) with electrooculography (EOG), electromyography (EMG), and relevant peripheral signals such as respiration, airflow, oximetry, and electrocardiography (EKG). These clinical sleep studies are read for sleep disorders (e.g., sleep apnea) and gross sleep architecture (e.g., time in cycling sleep stages of non-rapid-eye-movement (NREM) and rapid eye movement (REM) sleep. However, these studies rarely speak to the relevant specific biology of these states.

## The regulation of sleep

Sleep is regulated by two processes [1]. The circadian timing system—Process C—favors wake during the day and sleep at night. It achieves entrainment through light-sensitive retinal ganglia projecting to the hypothalamic suprachiasmatic nucleus, in turn regulating pineal melatonin release to facilitate sleep. Alterations to light-dark, or sleep, timing (e.g., shift-work, travel) lead to dysregulation and “jetlag.” In parallel, homeostatic sleep need—Process S—increases across wake and decreases across sleep, regulated in part by basal forebrain adenosine. Naps prematurely discharge sleep need, late waking can short-change it, and adenosine antagonists (caffeine) can mask it—compromising nocturnal sleep. Conversely, extended wakefulness can lead to catastrophic sleepiness, overriding circadian drives for alertness. Healthy sleep requires both processes in alignment.

## The neurobiology of sleep

**Fig. 1:**
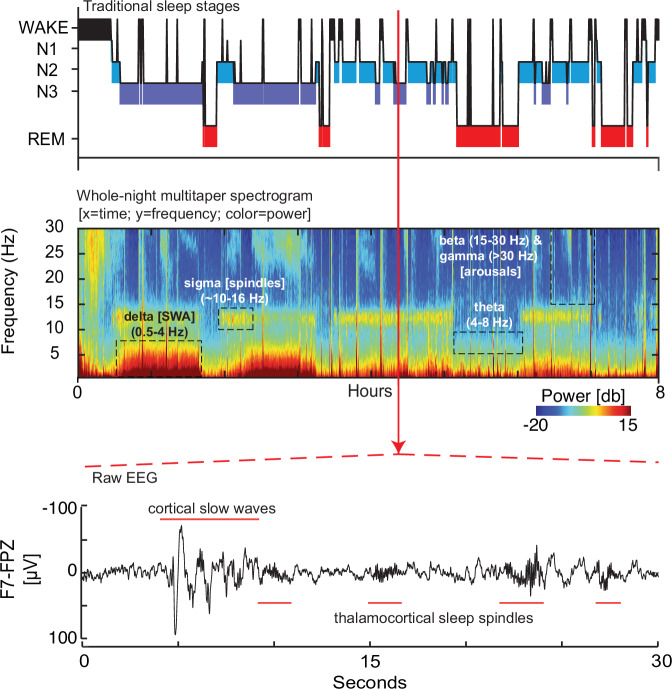
Highlighting sleep neurophysiology in a psychiatric context. This figure illustrates the richness of sleep neurophysiology beyond that typically acquired in a clinical sleep study. De-identified data are plotted from a night of at-home prefrontal sleep EEG recorded using a 2-electrode wearable headband (ZMax, Hypnodyne, Inc.) acquired from an adolescent research participant with complex psychiatric comorbidities (following informed consent). (**Top panel**): traditional sleep hypnogram progressing through 3 stages of NREM sleep (N1, N2, N3 [e.g., slow wave sleep] in increasing depth and REM sleep across multiple ultradian cycles. (**Middle panel**): Sleep spectrogram. This time-frequency decomposition of the whole-night EEG using multi-taper spectral analysis reveals a complex multi-dimensional neurophysiological milieu. The y-axis indicates frequency components of the EEG. The x-axis indicates time. Color indicates EEG power spectral density at each frequency-time pair on a decibel (10*log10) scale. Thus, each column of the spectrogram reveals the full power spectrum in a snapshot in time (e.g., one 30 s epoch); each row captures how a single frequency bin varies in power over time. Thus, the spectrogram reveals the full complexity of the time-frequency nature of the EEG. Close examination will reveal cyclic activity reflecting heightened delta (i.e., slow wave activity; SWA;0.5 - 4 Hz) and sleep spindle-related sigma activity (10–16 Hz) in NREM sleep, as well as relative increases in theta activity (4–8 Hz) in REM sleep. Arousals and transitions to wakefulness are reflected by increased power in higher frequency bands. (**Bottom panel**): One 30 s epoch of continuous EEG (in microvolts [μV]) extracted from NREM sleep illustrates the expression of both slow wave (≤2 Hz; ≥75μV) and sleep spindle (10–16 Hz) oscillations; parameters from these oscillations can be quantified and collated across sleep stages, time, and subject populations (relevant variables are expressed in the text and in the **glossary**).

Human sleep is characterized by repeating cycles of sleep stages typically summarized in a hypnogram (Fig. 1**; top panel**). NREM sleep consists of progressively quieter aminergic tone across three descending states of arousal (N1, N2, and N3 sleep) as a result of GABAergic modulation from sleep-promoting hypothalamic nuclei. REM sleep, conversely, results from cholinergic brain-stem circuitry silencing aminergic tone. These distinct neuromodulators yield electroencephalographic features (Fig. 1**; middle-bottom panels)** relevant to psychiatric and neurodevelopmental populations:

NREM Slow waves: Deep NREM slow wave sleep (N3) is named for its synchronized oscillations (typically ≤2 Hz) reflecting hyperpolarization and depolarization of cortical pyramidal cells. Slow waves index sleep homeostatic regulation as well as synaptic plasticity; their amplitude and incidence decrease across the night and are potentiated after time awake. Experimental challenges (e.g., wake extension) reveal the health of this homeostasis. Slow waves can be quantified by their magnitude (amplitude), density (rate), speed (intrinsic frequency), and morphology (e.g., slope) as well as their topographic expression. EEG power in the delta band (e.g., 0.5 – 4 Hz) has been deemed “slow wave activity” (SWA) and tracks these properties. SWA has been implicated in such conditions as mood disorders (e.g., altered SWA dissipation in major depression [2]).

NREM Sleep spindles: Nested in the depolarized up-state of cortical slow oscillations are short (~1–2 s) phasic bursts of sigma activity (~10–16 Hz). These “sleep spindles” join K-Complexes (brief high-amplitude waveforms of sequenced hyper- and de-polarization) as hallmarks of N2 sleep. Sleep spindles are paced by reciprocal interactions of the thalamic reticular nucleus and thalamocortical neurons and long been associated with sensory gating, consolidating sleep. More recently, their tight coupling to excitatory cortical up-states has been implicated in trait (e.g., IQ) and state (e.g., sleep-dependent memory consolidation) cognition. Spindle markers relevant to psychiatric disorders include density (occurrence rate), amplitude, intrinsic frequency, duration, and the magnitude and peak phase of coupling with slow waves [3].

REM Sleep microfeatures: REM sleep through its cholinergic cortical activation is characterized by desynchronized low voltage mixed-frequency EEG. REM can be quantified through rapid eye movement density (# eye movements / minute), latency of REM sleep onset, and power in relevant EEG bands (e.g., theta (4–8 Hz), beta (15–30 Hz), gamma (>30 Hz)). Decreased REM density, shortened REM sleep onset latency and high-frequency EEG activity have been associated with altered affective processes, and worse mood/anxiety symptoms [4].

**Glossary:** Sleep neurobiology relevant to psychiatric disorders research and treatment.

	Neurobiology	Function	Example variables	Clinical relevance
**Sleep-wake regulation**				
Sleep homeostasis (Process S)	Basal forebrain adenosine	Regulate sleep need and sleep pressure.	Sleep onset latency Time course of slow waves at night and after wake extension	Depression
**Key neurophysiological markers**				
NREM Slow waves	Synchronized oscillations during deep NREM sleep (e.g., N3). Indicating cortical up-down states driven by GABAergic inhibition of cortex	Sleep homeostasis Synaptic plasticity Development and aging	EEG delta power (0.5-4 Hz, i.e., slow wave activity). Some studies subdivide delta to slow (≤ 1 [or 2] Hz slow oscillations vs. 2-4 Hz faster delta), Individual slow wave (typically ≤ 2 Hz) amplitude, density, slope, intrinsic frequency, topography.	Depression, anxiety, ADHD
NREM Sleep Spindles	Phasic events (2 s; 10-16 Hz) during NREM sleep (N2 dominant) resulting from reciprocal interactions of the thalamic reticular nucleus and thalamocortical neurons.	Sensory gating Cognition (IQ, sleep-dependent memory) Cortical plasticity	EEG sigma (10-16 Hz) power Spindle density, amplitude, duration, frequency, coherence	Schizophrenia, autism, ADHD, anxiety
REM sleep microfeatures	Cholinergic cortical activation by pontine brainstem nuclei	Emotional regulation Arousal Dreaming and creativity	Mixed frequency EEG: including increases in theta (4-8 Hz) and high-frequency (e.g., beta [15-30 Hz] or gamma [> 30 Hz]) power Saw-tooth waves REM sleep onset latency Rapid eye movement density (#/minute) Periods of phasic (with rapid eye movements) vs. tonic (without rapid eye movements) REM sleep.	Depression, PTSD, anxiety disorders

## Improving measurement in a psychiatric setting

Clinical sleep studies stop short of the oscillatory features enumerated above. For example, an individual with 75 μV amplitude slow waves and another with 250 μV may have equivalent N3 sleep time. An individual with one spindle every three minutes may have the same N2 sleep time as one with three spindles every minute. This coarse lens limits utility of traditional sleep studies for psychiatric neuroscience. Open-source tools for advanced EEG analytics (including post-hoc analysis of clinical studies) have become widespread, creating new opportunities to learn from these mechanistically rich data. Even with tools in hand, traditional sleep studies’ instrumentation poses rate-limiting burdens on psychiatric or neurodevelopmental populations. While still needing validation (e.g., in developmental and patient groups), scalable and wearable sleep EEG (e.g., headbands, patches) may overcome some barriers of measurement in psychiatric contexts.

## Do not neglect circadian biology

Our primer focuses on sleep neurophysiology; however, psychiatry professionals should not dismiss circadian biology. Circadian rhythms can be characterized by subjective reports (e.g., chronotype and morningness/eveningness), activity rhythms (e.g., acrophase and amplitude, intradaily variability and interdaily stability), and biomarkers (e.g., dim-light melatonin profiles, core body temperature). Blunted rhythmicity as well as phase-shifted or misaligned rhythms are present, for example, in mood disorders [5].

## Considerations for interested clinicians

Sleep is closely connected to mental health. Whether through psychotherapy, light-based chronotherapy, or neuromodulatory interventions targeting sleep may be impactful for individuals suffering from psychiatric symptoms (e.g., [6]). Gaining familiarity with the neurophysiology and biology of sleep, and relevant measurement options may allow psychiatry professionals to integrate targeted and adjunctive treatments focused on sleep. In addition, close monitoring of sleep may serve as an indicator of early treatment response, as healthy sleep can support improved cognitive and affective functioning. More broadly, integrating sleep neuroscience into psychiatric research and practice will benefit both mechanistic discoveries and identification of actionable treatment targets.

## Conclusions

Sleep is a deceptively simple behavior that manifests from complex biological processes. We hope that this primer serves to introduce some of the core neuroscientific concepts underlying sleep regulation, the physiological markers measurable in the sleeping brain, and their psychiatric relevance.

## Data Availability

Figure 1 represents data from an adolescent research participant. For didactic purposes, code used to recreate the analyses of Fig. 1 are available upon request to the authors. Example data (including non-research mock datasets) may be subject to institutional data sharing requirements.
